# A Joint Less Ordinary: Intriguing Roles for Hedgehog Signalling in the Development of the Temporomandibular Synovial Joint

**DOI:** 10.3390/jdb4030025

**Published:** 2016-08-26

**Authors:** Malgorzata Kubiak, Mark Ditzel

**Affiliations:** Edinburgh CRUK Cancer Research Centre, MRC Institute of Genetics and Molecular Medicine at the University of Edinburgh, Edinburgh EH4 2XR, UK; malgosia.m.kubiak@gmail.com

**Keywords:** Hedgehog, Hh, Indian Hedgehog, IHH, temporomandibular joint, synovial joint, GLI

## Abstract

This review highlights the essential role of Hedgehog (Hh) signalling in the developmental steps of temporomandibular joint (TMJ) formation. We review evidence for intra- and potentially inter-tissue Hh signaling as well as Glioma-Associated Oncogene Homolog (GLI) dependent and independent functions. Morphogenesis and maturation of the TMJ’s individual components and the general landscape of Hh signalling is also covered. Comparison of the appendicular knee and axial TMJ also reveals interesting differences and similarities in their mechanisms of development, chondrogenesis and reliance on Hh signalling.

## 1. Joint Types: An Overview

A joint is an essential skeletal element that is defined as a place where two or more bones meet and form a connection. This connection then provides a degree of mechanical support and in some cases the ability to articulate. The distinct joints of the axial and appendicular skeleton can be classified by different criteria.

Criteria (1) Range of motion:

(A) Immovable, such as the fibrous skull sutures.
(B) Capable of limited movement, such as the cartilaginous pubic symphysis.
(C) Full movement, such as the synovial knee and temporomandibular joints.

Criteria (2) Structural composition of the connective tissues:

(A) Fibrous joints that unite through thick connective tissue, such as the interosseous membrane between the tibia and fibula.
(B) Cartilaginous joints that are united through cartilage, such as the pubic symphysis.
(C) Synovial joints that have an articular capsule between two interconnected bones, which are themselves protected by articular cartilage and frequently reinforced by ligaments. These synovial joints can be further subdivided into:
  (i) Gliding joints that articulate on a single plane (e.g., intervertebral joints).
  (ii) Hinge joints move on a single axis (e.g., knee joint).
  (iii) Hinge and sliding joints that articulate on a single plane and axis (e.g., temporomandibular joint).
  (iv) Pivot joints provide rotation (e.g., atlas and axis joint).
  (v) Condyloid joints permit circular motions, extension and flexion (e.g., radiocarpal joint).
  (vi) Saddle joints allow extension and flexion, but no rotation (e.g., carpometacarpal joint).
  (vii) Ball and socket joints exhibit free movement on any axis (e.g., shoulder joint).

**Figure 1:**
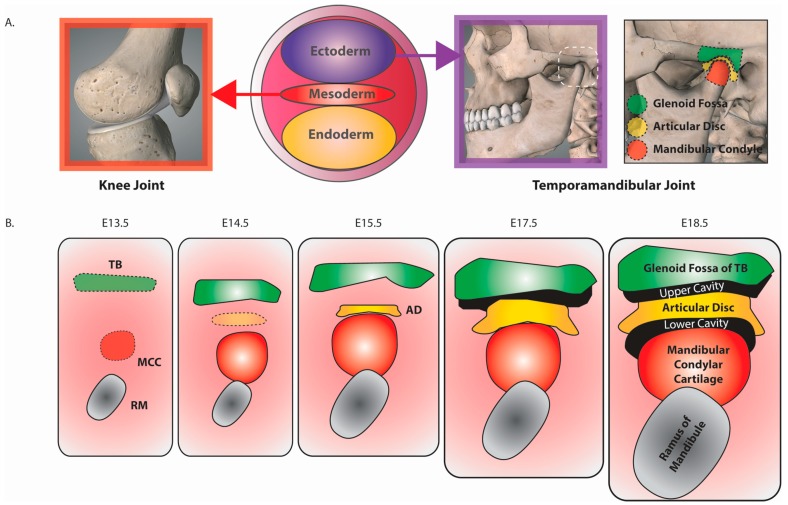
Embryonic origins and development of the temporomandibular joint. (**A**) Appendicular joints like the knee joint are derived from the embryonic mesoderm, whereas the axial temporomandibular joint is derived from the embryonic ectoderm. The boxed region is enlarged to show the location of the mandibular condyle (MC), articular disc (AD) and glenoid fossa (GF). It also corresponds to the developing tissues indicated in (**B**). Please note that the MC contains the cartilaginous region termed the mandibular condyle cartilage (MCC); (**B**) Development of components of the murine temporomandibular joint (TMJ). Approximate embryonic stages are indicated above each panel. This model indicates independent mesenchymal condensations for the temporal bone (TB), mandibular condylar cartilage (MCC) and articular disc (AD)—please see text for controversy concerning the developmental origins of the MCC. Labelled components of the TMJ are indicated at E18.5. Please note that the temporal bone contains the subregion termed the glenoid fossa (GF) and TMJ development continues postnatally (not shown). The capsule that envelops the joint and one major and two minor ligaments associated with the TMJ are not depicted. Figure adapted from Suzuki et al. [8].

These various joint types can arise from different embryonic origins. For example, the knee joint is mesodermally derived, while those of the temporomandibular joint are ectodermally derived (Figure 1A). The majority of joints initially develop from mesenchymal condensations that in turn undergo distinct developmental processes. For example, in fibrous and cartilaginous joints, the mesenchyme between developing bones (aka interzone) differentiates [1,2] into dense fibrous or hyaline/fibrocartilage, respectively. In synovial joints, the outer mesenchyme forms the joint capsule and ligaments, while the central mesenchyme is removed to form the synovial cavities [3]. Mesenchyme lining the joint capsule and articular surfaces of the bones then go on to form the synovial membrane. Hence, the interzone cells of the developing synovial joint contribute to all of the components of the joint [1,4].

Representing the less famous relative of the knee joint, the TMJ exhibits a number of distinctive developmental steps that are regulated by Hh signalling. As found in the joints of the long bones [5,6], Hh signalling is essential for correct TMJ development, growth and maturation. However, clear phenotypic differences exist between different Hh-pathway-mutant animals. These observations therefore identify potentially divergent roles for different Hh pathway effectors in distinct aspects of TMJ development. Please note that a number of excellent reviews cover broader aspects of TMJ development in more detail [7,8], whereas this review specifically focuses on the role of Hh signalling.

## 2. The Temporomandibular Joint

The TMJ is a synovial joint of the axial skeleton involved in the hinge- and sliding-articulation of the jaw (Figure 1A). Its major components include (i) the mandibular condyle (MC), articular disc (AD), glenoid fossa (GF) of the temporal bone (TB), upper and lower synovial cavities (Figure 1B) and the ligamentous structures of the joint capsule and lateral, sphenomandibular and stylomandibular ligaments. Acting together, these components provide a limited degree of movement of the GF and MC against the AD to facilitate hinge- and gliding-joint function. This action then facilitates, amongst others, the acts of mastication, speech, swallowing and laughter. Developmental disorders [9] and diseases such as osteoarthritis [10] that impacts upon TMJ function therefore affects many important aspects of life.

The TMJ is of particular interest as its structure, composition and development differs significantly from that of the knee synovial joint. In contrast to the knee joint in which bones are separated by a single synovial space, the articular surfaces of GF and MC are separated by the fibrous AD and an upper and lower synovial space (Figure 1B). The fibrous cartilage of the AD and that located on the articulating ends of the MC and GF differ from the hyaline cartilage of the knee joint. The superficial layers of the MC and GF abutting the AD comprise of an articular perichondrium of relatively undifferentiated cells that express Collagen type I alpha 1 chain (COL1A1), rather than the Collagen type II alpha 1 chain (COL2A1) expressing chondrocytes of the hyaline articular cartilage [11]. Furthermore, the cartilaginous element of the MC, known as the MC cartilage (MCC), is classified as secondary cartilage [12]. In contrast to the primary cartilage of the knee joint, the components of the TMJ are derived independently of the chondroskeleton [13]. Rather than the mesodermal origins of the chondroskeleton (e.g., knee joint), the MCC is derived from the ectodermal neural crest and develops relatively late during embryonic development [14].

## 3. Murine TMJ Development

Joints can differ in their mechanism of development. In the case of the knee joint, a single mesenchymal condensation is subsequently subdivided into the joint’s distinctive components [1,2]. In contrast, distinct mesenchymal condensations give rise to TMJ components [15]. While the AD and TB are derived from individual condensations, there is some controversy over whether the MCC derives from a distinct mesenchymal condensation or via a periosteal region of the ramus of the mandible [16]. Nevertheless, as the mandible itself arises from a mesenchymal condensation it is true to say that each of the three TMJ components arise from distinct mesenchymal condensations. For the purpose of this review we are indicating individual mesenchymal condensations for the AD, MC and TB. Once formed each of the components coalesce to form the TMJ (Figure 1B) [7,8].

In the mouse, the precursor mesenchymal condensations of the temporal bone and MCC form first around E13.5 (Figure 1B). The AD precursor mesenchymal condensation then forms in-between those of the TB and MC at around E15.5. Formation of both upper and lower cavities is readily detectable by E18.5, as is expression of the joint lubricating substances *Lubricin* on the articular surfaces of the MC, AD and GF [17]. Interestingly, the growth differentiation factors (GDFs) proteins essential for knee joint cavitation [1,2] are not expressed in the TMJ interzone mesenchyme [17]; therefore, indicating quite a substantial difference in the molecular mechanism involved in promoting joint cavitation.

**Figure 2:**
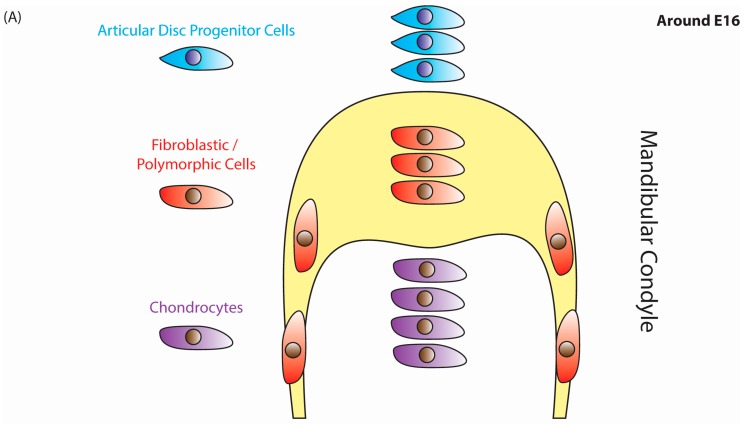

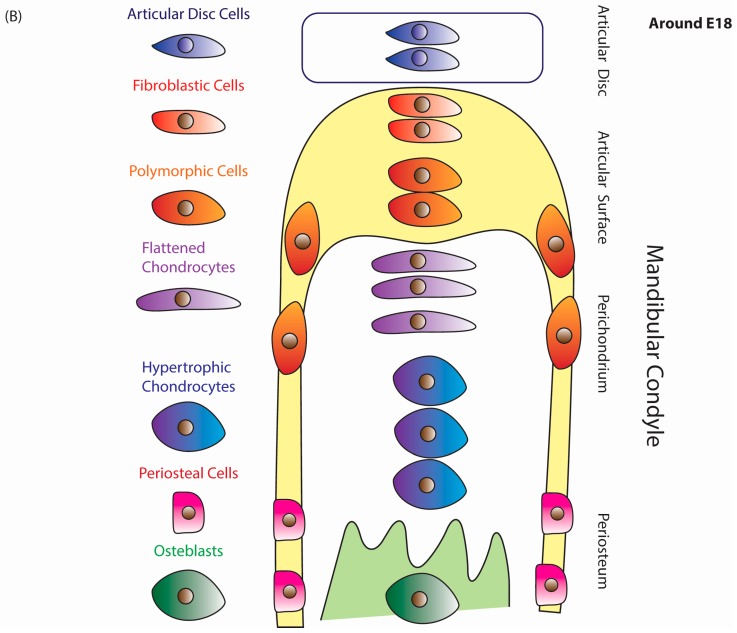
Cell profile during mandibular condyle and articular disc development. (**A**) Schematic of cells types within a developing mandibular condyle and the mesenchymal precursors of the articular disc around E15. The articular disc proper has not yet formed. The anterior end of the mandibular condyle harbours a layer of fibroblastic and polymorphic cell types that overlay an inner mass of chondrocytes. Above them lies a group of mesenchymal articular disc precursor cells; (**B**) Around E18 articular disc development has initiated. Cells within the anterior region of the mandibular condyle have separated into the anterior most fibrous cell layer and the underlying polymorphic layer. These perichondrial layers surround flattened chondrocytes and more posteriorly located hypertrophic chondrocytes. Posterior to these lies the region undergoing endochondral ossification that harbours osteoblasts and trabecular bone (light green region) that are flanked by the periosteum. Figure adapted from Shibukawa et al. [32]. The transition from a perichondrium into a periosteum also marks the interface between the MCC and ramus of the mandible.

The MC itself appears central to both TMJ and facial development, orchestrating tissue growth of the mandible, AD, GF as well as other tissues of the midface [18]. MC development is of particular interest as the chondrogenic MCC arises adjacent to a bone, the ramus of the mandible, undergoing intramembranous ossification—a process whereby bone is formed independent of a cartilage intermediate [19]. These two tissues exhibit a continuous transition from the posterior localised osteogenic periosteum of the ramus into the anterior-localised chondrogenic perichondrium of the MC (Figure 2). A similar situation is also observed along peripheral layers of the long bone as the articular cartilage undergoes a transition into perichondrium and subsequently a periosteum [20].

The chondrogenic perichondrium of the MCC harbours undifferentiated, polymorphic, prechondroblastic cells that secrete COL1A1 [11]. This is in stark contrast to the COL2A1 expressing chondrocytes within growth plates of long bones or articular cartilage of the knee joint [21]. The ability of the MCC’s peripherally located prechondroblastic cells to both proliferate and differentiate into chondrocytes provides them with the ability to drive appositional growth [22]. This form of growth is in contrast to the interstitial growth observed within the growth plate of long bones [23,24], but like the growth observed in the articular cartilage of the knee joint [25]. Unlike the chondrocyte-directed growth in long bones and its associated articular cartilage, non-chondrocyte-like prechondroblastic cells mediate growth in the MCC. Therefore, it is a chondroprogenitor cell population that undergoes proliferation and chondrogenesis to produce chondrocytes. These chondrocytes will then be incorporated into the underlying cartilage [26] and subsequently undergo maturation and hypertrophy. This chondrogenic growth and subsequent endochondral-mediated ossification thereby promotes the growth of the MCC and its intercalation with the ramus of the mandible [27].

Intriguingly, the chondroprogenitor polymorphic cells of the MCC have the ability to promote either bone or cartilage in response to different mechanical stimuli [28,29]. Hence, these polymorphic cells can be classified as dual chondro- and osteo-progenitor cells, a classification that is supported by their expression of both chondrogenic-associated *Sry-related HMG box 9* (*Sox9*) and osteogenic-associated *Runt-related transcription factor 2* (*Runx2*) [30]. In contrast, *Runx2* positive hypertrophic and *Sox9* positive non-hypertrophic chondrocytes within primary-cartilage growth plates and hyaline articular cartilage exhibit mutually exclusive expression patterns [31].

**Figure 3:**
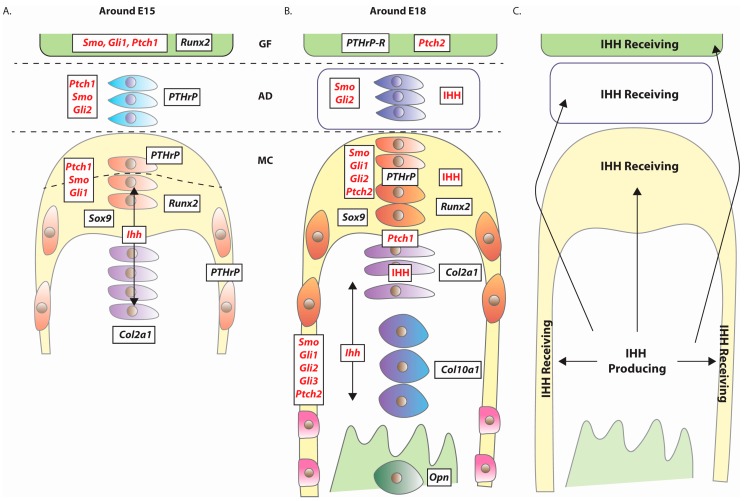
Hh signaling within the developing TMJ. Approximate representation of the murine expression patterns of various chondrogenic and osteogenic regulators (black text) including components of the Hh signalling pathway (red text) around (**A**) E15 and (**B**) E18. The bulk of supporting data is from in situ hybridisation studies of mRNA expression [17,32,34], with only IHH ligand protein expression established in E18 tissue [33]. Please note that IHH is an extracellular signalling molecule capable of long-range transport, meaning that IHH protein does not necessarily have to co-localise with those cells expressing *Ihh* (e.g., the detection of IHH protein in non-*Ihh* expressing fibrous cells and articular disc cells). Such observations support a role for intra- and inter-tissue IHH-mediated signalling. (**C**) A generalised overview of the regions initiating Hh signalling (*Ihh* expressing), responding to and/or capable of being stimulated by IHH ligand. Figure adapted from Shibukawa et al. [32].

By E17.5-E18.5, the MCC exhibits a growth-plate-like structure consisting of four cellular zones (Figure 2B), namely the peripherally located *Sox9* and *Runx2* expressing perichondrial layers of fibrous and polymorphic-progenitor cell layers [17,32,33,34], which in turn overlay *Col2a1* positive flattened chondrocytes and *Collagen type X alpha 1 chain* (*Col10a1*) positive hypertrophic chondrocytes [35] (Figure 3). Although differing in cellular composition and structure, the growth plate of the long bones and growth plate-like structure of the MCC are functionally equivalent as a means for mediating tissue growth.

## 4. Expression of Hedgehog Pathway Components

Hedgehog (Hh) signaling plays essential roles in animal development and homeostasis [36]. It normally functions in a paracrine manner as an extracellular signaling pathway, with one cell producing the Hh ligand and another cell perceiving the ligand. However, in some situations autocrine signaling may also occur whereby the Hh ligand is both produced and perceived by the same cell. In skeletal development, two Hh family members play important roles, with Sonic Hedgehog primarily governing early limb patterning [37] and Indian Hedgehog (IHH) controlling cell proliferation and differentiation later on during embryonic development [38,39]. For more detailed information about Hedgehog pathway signal transduction, please see Alman et al. [5].

During murine development, *Ihh* is expressed throughout TMJ development, from its earliest inception through to embryonic and postnatal development [17,30,32,33,34,40]. *Ihh* is expressed in the MCC-associated E13.5 mesenchymal condensations and continues to be expressed in early chondrocytes at E15.5 (Figure 3A). Pre-hypertrophic and hypertrophic chondrocytes continue to express *Ihh* at E18.5 (Figure 3B). Although *Ihh* is not expressed in the E15 embryonic GF [34], recent work in the postnatal TMJ revealed *Ihh* expression within a one-month-old glenoid fossa [41].

A number of mouse models, described in more detail below, strongly indicate MCC-derived IHH as a central coordinator of cells both within the MCC as well as those in the surrounding tissues. These IHH-mediated effects, as in the growth plate of primary cartilage [5], most likely act in a juxtacrine/paracrine manner to promote cell proliferation and differentiation.

Around E15.5, the expression of one of IHH’s cognate receptors, *Patched1* (*Ptch1*) (Figure 3) [17,32,33,34], indicates the cells’ potential to perceive and respond to extracellular IHH ligand. Such cells include (i) flattened chondrocytes; (ii) disc progenitor cells within the mesenchyme surrounding the articular surface of the MC and (iii) cells within the GF. By E18.5, *Ptch1* expressing cells appeared to be restricted to those around the interface between the flattened chondrocytes and the polymorphic cells layers. Another IHH receptor, *Ptch2*, was expressed within the polymorphic cell layer and GF [17]. Activation of Hh signalling was detected in the postnatal MCC by expression of *Gli1*-*LacZ* Hh reporter in the polymorphic cells, flattened chondrocytes and AD cells [40].

To summarise, in earlier embryonic stages *Ihh* is primarily expressed by early chondrocytes, and later on by flattened and hypertrophic chondrocytes [17,32,33,34]. In general, *Ptch1* and *Ptch2* expressing cells either surrounded *Ihh* expressing cells or were present in other non-*Ihh*-expressing tissues (i.e., AD and GF) (Figure 3A,B). Interestingly, the chondrocytes cells within the early E15.5 MCC appeared to have the potential to both express *Ihh* and respond to IHH. This scenario would permit autocrine and/or localized paracrine signalling between similar cell types within the flattened chondrocyte cell layer. Overall, IHH signalling appeared to potentially act over a short range within the MCC and at a longer range between the MC and the AD and GF (Figure 3C). This ability of IHH to signal between tissues is supported by *Drosophila* Hh’s ability to be packaged into exosomes [42,43] and be delivered across extracellular spaces via filopodia-like extensions called cytonemes [44,45]. Furthermore, *Drosophila* Hh can act as an endocrine factor between the intestine and the fat body [46].

In the postnatal TMJ, however, the likelihood of MC-derived IHH signalling to the GF is reduced as chondrocytes within the GF itself expresses *Ihh* [41]. Therefore, in one-month old GF, the IHH detectable in the GF [41] is more likely to be GF-derived than MC-derived.

## 5. TMJ Defects Associated with Loss of Hedgehog Signalling

### 5.1. Introduction

The clearest example of the importance of Hh signaling in TMJ development comes from observations of germline loss of *Ihh* function. *Ihh* null TMJ exhibit a number of defects, described in more detail below, that include shortened mandibles, reduced size of the MC, cellular disorganisation of the MCC and absence of the AD and lower articular cavity [32].

Mechanistically, these defects are believed to be due to reduced activity of canonical Hh signaling—please see Alman et al. [5] for more details. The loss of IHH ligand production would lead to (i) repression of Smoothened (Smo) signalling, the Hh pathway’s key positive regulator and G-protein coupled receptor, which in turn would result in (ii) increased proteolytic processing of Glioma-Associated Oncogene Homolog 3 (GLI3) into a transcriptional repressor (GLI3R) and (iii) decreased activation of the transcriptional activity of Glioma-Associated Oncogene Homolog 2 (GLI2) [47]. Hence, loss of *Ihh* function would be predicted to suppress the transcriptional outputs of the Hh pathway through increased GLI3R-mediated transcriptional suppression and decreased GLI2-mediated transcriptional activation of Hh pathway target genes.

Prior to describing the results of disrupting Hh signaling components on TMJ development, it is important to bear in mind that different mouse models utilised distinct approaches to disrupt gene function. While some used germline nulls that resulted in embryo-wide loss of gene function from the point of conception, others utilised Cre-mediated recombination to lose gene function in a spatio-temporal restricted manner. For example, *Wingless/Integrated 1-* (*Wnt 1)*, *Col2a1*- or *Sox9*-Cre driver lines combined with flanking ‘*locus of* *X-over P1*’ (*loxP*) alleles (known as a ‘*floxed*’ or ‘*fl*’ allele) permitted conditional loss of gene function within certain chondrogenic-associated tissues at certain times in their genesis. Therefore, it is important to consider the method of gene loss when comparing phenotypes. Below we briefly summarise the common Cre-driver lines used to study TMJ development:

A. *Wnt1*-Cre leads to effective loss of gene function in neural crest cells prior to formation of the TMJ-associated mesenchymal condensations. Hence, *Wnt1-Cre* [48] deletes gene function very early on in TMJ developmental precursors [34] and would be predicted to create an entirely mutant TMJ. 

B. Within the MCC, *Col2a1* is strongly expressed in flattened and hypertrophic chondrocytes, a sublayer of polymorphic cells immediately adjacent to the flattened chondrocytes and within the periosteum (Figure 3A,B). *Col2a1* is not expressed within the fibrous cell layer [49]. Hence, *Col2a1*-Cre expression would predominantly mediate recombination in distinct regions of the MC and produce chimeric tissue for the *floxed* gene of interest.

C. *Sox9* is expressed in the early TMJ-associated mesenchymal condensations [50], with *Sox9*-Cre therefore mediating loss of the *floxed* gene of interest very early in TMJ development. As with *Wnt1*-Cre, such a system would be predicted to create an entirely mutant TMJ.

### 5.2. Mandibular Condyle Development

#### 5.2.1. Indian Hedgehog (*Ihh*)

At E17.5, germline *Ihh* null mandibles were smaller than normal and exhibited a reduction in bone formation and a concomitant increase in the amount of cartilage [32]. Postnatally, the scenario reversed with the P0 mandible exhibiting extensive ossification at the expense of the cartilage at the condylar process. In a normal MC, *Col10a1* and *Osteopontin* (*Opn*) are two key players that promote ossification [51]. Their expression patterns are predominantly mutually exclusive within the posterior for the MC, with a small overlap at the interface between hypertrophic chondrocytes and the newly forming bone [32] (see Figure 3B). In *Ihh* null animals, rather than being localised within the posterior half of the MCC, *Col10a1* was expressed around the peripheral interface between the inner mass of hypertrophic chondrocytes and outer layer of polymorphic cells [32]. Intriguingly *Col10a1*, a marker associated with hypertrophic chondrocyte function, did not overlap with the bulk of the hypertrophic chondrocytes within the core of the *Ihh* null MCC. Therefore, the cells that appeared to be morphologically similar to hypertrophic chondrocytes were not functionally equivalent.

In *Ihh* nulls *Opn* was also spatially misexpressed across the entire inner mass of hypertrophic chondrocytes [32]. The combined misexpression of *Col10a1* and *Opn* may have accounted for the TMJ ossification defects observed in *Ihh* null MCC. As in the long bones [5], *Ihh* plays an important role in regulating the balance between chondrogenesis and ossification in the TMJ. The reduced size of the *Ihh* null mandible was potentially explained by (i) the decreased cellular proliferation within the fibrous and polymorphic layers; (ii) a massive increase in hypertrophic chondrocytes and (iii) a dramatic reduction in flattened chondrocytes. Taken together, these results suggested that the MCC had potentially undergone (A) an increased rate of differentiation of the flattened chondrocytes into hypertrophic chondrocytes, and/or (B) a reduction in the rate of production of flattened chondrocytes by the fibroblastic and polymorphic chondroprogenitor layers. Such cellular defects would be predicted to have reduced the chondrogenic potential of the MC and hence its growth potential. In agreement with the reduction in size of the fibroblastic/polymorphic chondroprogenitor domain, *Ihh* null animals exhibited a reduction in the expression level of chondrogenic *Sox9* [17,32] and its downstream effectors *Sox5* and *Sox6* [17]. Hence, *Ihh* appeared important for the maintenance of the chondrogenic potential of the MC and its ability to increase in size.

Expression of *Parathyroid hormone-related protein* (*PTHrP*), a potent regulator of chondrocyte behavior intimately linked to Hh signaling [52], was totally absent from the perichondrium of the *Ihh* null MCC [32]. Within the developing long bone, IHH signals to the articular perichondrium to express *PTHrP*/PTHrP, which in turn suppresses the differentiation of chondrocytes into pre-hypertrophic chondrocytes [52]. In a potentially analogous situation, chondrocyte-derived IHH may promote *PTHrP/*PTHrP expression in fibrous/polymorphic cells of the MCC, that in turn signal back to the PTHrP-receptor (PTHrP-R) expressing polymorphic cells at the interface with the flattened chondrocytes (Figure 3A,B). PTHrP-mediated signalling to these polymorphic chondroprogenitor cells may then promote their proliferation and restrain their ability to differentiate into flattened chondrocytes. Expression analysis also revealed the possibility for inter-tissue signalling between *PTHrP* expressing MCC- and AD-resident cells with *PTHrP-R* expressing cells in the GF.

In long bones, IHH can also function independently of PTHrP to promote chondrocyte proliferation [53] and formation of flattened columnar chondrocytes within the growth plate [54]. Hence, the reduction in the number of flattened chondrocytes could be attributed to a similar role for IHH in the MCC, whereby it would (i) directly promote proliferation of flattened chondrocytes and/or (ii) promote the proliferation of the polymorphic chondroprogenitor cells and/or their production of flattened chondrocytes. In summary, the loss of both IHH and/or PTHrP expression most likely accounted for the reduced size and chondrogenic potential of *Ihh* null MC.

Similar to germline loss of *Ihh*, *Col2a1*-Cre-ER-mediated postnatal loss of *Ihh* revealed disruption of the cell layers within the MCC. There was a marked reduction in the size of the polymorphic and flattened chondrocyte zones, an absence of proliferation in the polymorphic layer and mixing of *Col2a1* and *Col10a1* expression domains. The MC also harboured ectopic bone marrow cavities, reduced trabecular bone volume and spatially misplaced hypertrophic-like chondrocytes. Expression of *Sox9*, *Col2a1* and *Runx2* were also significantly reduced in the *Ihh*-deficient MCC [40]. Overall, these results supported an important role for *Ihh* in influencing TMJ-associated postnatal chondrogenesis and development.

A recent study identified the effects of *Wnt1-*Cre-mediated *Ihh* overexpression (*Ihh^OE^*) on TMJ development [55]. These *Ihh^OE^* animals exhibited craniofacial abnormalities including TMJ defects. Intriguingly, the glenoid fossa, which along with the MCC is derived from *Wnt1* expressing cranial neural crest progenitors, failed to develop. At E14.5, the mesenchymal condensation of the GF was absent, whereas the MCC condensation had formed and appeared normal. At E17.5, there was no sign of a GF, with only fragments of bone-like tissue present in the presumed position of the GF. In contrast, the MCC, AD and lower synovial cavity appeared to develop normally. Hence, these observations suggested that GF development was extremely sensitive to elevated levels and/or ectopic *Ihh* expression.

Closer examination of the E15.5 and E16.5 *Ihh^OE^* MCC revealed a number of cellular defects including apparent expansion of the polymorphic and flattened chondrocyte cell layers [55]. Such an expansion of chondrocytes and their polymorphic progenitor cells would be in agreement with a role for IHH in promoting non-hypertrophic chondrocyte proliferation and suppressing the production of hypertrophic chondrocytes. E16.5 molecular defects agreed with this model, with a corresponding increase in expression of the non-hypertrophic markers (*Sox9* and *Col2a1*) in the flattened chondrocyte and polymorphic cell layers. Evidence for perturbed chondrogenesis was also supported by increased *Runx2* expression in the perichondrial and polymorphic cell layers of E16.5 *Ihh^OE^* animals. Furthermore, the cellular domain not expressing the hypertrophic-associated marker *Col10a1* was increased. Together, these findings suggested an increase in the number of non-hypertrophic chondrocytes and their precursors. Such precursors may have included cells in the process of differentiating from polymorphic chondroprogenitors into flattened chondrocytes. Regardless of the actual cellular changes, it was clear that increased *Ihh* expression altered the magnitude and expression domains of key chondrogenic regulators.

Interestingly, the expression of the polymorphic chondroprogenitor cell marker *Col1a1* was decreased at the apical most region of the polymorphic layer [55]. Such a decrease in expression of a chondroprogenitor marker could be explained by a reduction in the polymorphic cell population, with stem/progenitor cell exhaustion [56] being a possibility. In such a situation, the polymorphic cells population would undergo precocious chondrocyte differentiation in the absence of polymorphic cell regeneration. Such actions would result in an increased rate of chondrocyte production along with a concomitant decrease in polymorphic cell numbers.

Taking into account the effects observed upon loss of *Ihh* function (see above), *Sox9* and *Runx2* within the polymorphic cell layers exhibited the expected opposing effects upon either gain [55] or loss of *Ihh* [32,40]. It therefore appeared to support the idea that IHH-mediated signalling promoted, either directly or indirectly, *Sox9* and *Runx2* expression within the perichondrial region. However, that simple relationship did not hold true when considering *Runx2* expression within the core of the MCC, whereby *Ihh^OE^* caused a dramatic loss of *Runx2* expression [55]. Only through more extensive and accurate cell type identification will we be able to establish whether changes in mRNA expression profiles reflect altered mRNA profiles (i) within the same cell types or (ii) between different cell types.

Regardless of the underlying cellular events responsible for the molecular defects, perturbing *Ihh* function clearly resulted in morphological defects that highlight its essential role in MCC development.

#### 5.2.2. Glioma-Associated Oncogene-2 (*Gli2*)

Mutations in the Hh pathway’s transcriptional effector human *GLI2* are associated with a range of human facial defects including mandibular hypoplasia [57]. Similar to *Ihh* nulls, germline loss of murine *Gli2* function using a DNA-binding deficient mutant allele resulted in a smaller MC at E14.5 [58], a disorganised MCC that exhibited increased numbers of hypertrophic-like chondrocytes and a decreased number of polymorphic and flattened chondrocyte cell layers at E16.5 and E18.5 [17]. 

The increase in hypertrophic-like chondrocytes in the *Gli2*-mutant MCC is proposed to be due to a loss of fibroblast growth factor signaling [59]. In agreement, perichondrial-associated *fibroblast growth factor receptor 2* (*Fgfr2)* and chondrocyte-associated *fibroblast growth factor receptor 3* (*Fgfr3)* expression was reduced in E18.5 *Gli2*-mutant animals [17]. Furthermore, *Fgfr3* null long bones phenocopied the *Gli2*-mutant associated expansion of hypertrophic cell zone [60]. Hence, these findings support the idea that FGF signaling suppresses the formation of hypertrophic chondrocytes.

*Fgfr3* is expressed primarily within the immature chondrocytes within the core of the E16.5 MCC with significantly weaker expression within the surrounding perichondrium [17,61]. In contrast, *Fgfr2* exhibits a more restricted expression pattern with signals solely detected in E16.5 MCC perichondrial regions [17,61]. The expression of *Gli1* and *Gli2* solely in the perichondrium [17] indicates (i) a direct, cell- and/or tissue-autonomous role for GLI-mediated Hedgehog signalling in regulating *Fgfr-2* and *-3* expression within the perichondrium and (ii) an indirect, non-cell-/non-tissue-autonomous role for GLI-mediated Hedgehog signalling in regulating *Fgfr3* expression within the central core of the MCC.

The reduction in expression of FGF receptors and a presumed concomitant decrease in FGF-mediated signalling may have contributed to the increased chondrocyte hypertrophy in *Gli2* mutants. As with the *Ihh* nulls, increased *Col10a1* expression was not apparent in the *Gli2 mutant* MCC. These data indicated that the hypertrophic-like chondrocytes were not expressing the expected hypertrophic gene markers. Additionally, *Gli2* mutant MCC exhibited a ‘hollowing out’ of the *Col10a1* signal within the MCC core, but retention of signal strength around the periphery.

Interestingly, germline loss of *Gli2* function failed to affect *Sox9* expression, despite a reduction in the size of the polymorphic cell layers [17]. Therefore, while loss of *Ihh* or *Gli2* resulted in a reduction in the cells associated with *Sox9* expression, only loss of *Ihh* actually resulted in a reduction in *Sox9* expression within those cells. These findings suggested that *Ihh*-mediated regulation of *Sox9* potentially occurred via a GLI2 independent pathway.

Another difference between loss of *Ihh* and *Gli2* function involved expression of *Gli3*. *Ihh* null MCC exhibited either no change or even a small reduction in the extent of the *Gli3* expression domain [32], whereas perichondrially located *Gli3* expression massively increased in *Gli2* mutant MCC [17]. These data suggested that GLI2 activity, potentially independent of IHH signaling, regulated *Gli3* transcription. Perichondrially located *Gli2* expression within wild-type MCC supports the possibility of a direct cell autonomous role for GLI2 in repressing *Gli3* expression. In summary, these findings highlight morphological similarities and molecular differences upon loss of an upstream (*Ihh*) and a downstream component (*Gli2*) of the Hh pathway.

#### 5.2.3. Indian Hedgehog (*Ihh*) and Glioma-Associated Oncogene-3 (*Gli3*)

To directly address a contribution of *Gli3* to the *Ihh* mutant phenotype, a loss of *Gli3* function was combined into an *Ihh* null background. The result was a partial rescue of the defects observed in the *Ihh* null MC [32]. The extent of the *Sox9* expression domain was rescued (increased) as was the proliferation of the fibrous and polymorphic cell layers (increased) [17]. In general, *Ihh* null MCC exhibited a dramatic loss of *Ptch1*, *Gli1* and *Gli2* expression and only minor differences in the spatial expression pattern of *Smo* and *Gli3*. Strikingly, *Ihh^−/−^*;*Gli3^−/−^* animals failed to re-express *Gli1* or *Gli2*. It therefore appeared that the lack of *Gli1/Gli2* expression observed in *Ihh* nulls was not due to GLI3R-mediated transcriptional repression. Yet, from the *Gli2* mutant studies, it appeared that GLI2 was involved in suppressing *Gli3* expression (see above).

In contrast to the Hh signalling landscape, the expression domains of *PTHrP* and *PTHrP-R* were more effectively rescued, albeit showing minor spatial misexpression. *Col10a1* was also spatially misexpressed, with its normal posterior expression domain shifted into the anterior domain. Interestingly, there was no effect on the ectopic MCC-wide expression of osteogenic-associated *Opn*.

In summary, these results clearly demonstrated that loss of *Gli3* function did not fully rescue the Hh-pathway molecular defects associated with loss of *Ihh* function.

#### 5.2.4. Smoothened (*Smo*)

To investigate the role of *Smo* in TMJ development, *Sox9*-Cre-mediated recombination [62] was used to delete *Smo* function in most of the cells within the MCC, including polymorphic cells [17]. Similar to the *Gli2* and *Ihh* null phenotypes, the *Smo*-deficient MC was smaller, exhibited a reduced flattened chondrocyte domain and increased hypertrophic chondrocyte domain [17]. Unfortunately, a lack mRNA expression analysis precluded determination of the Hh signalling landscape within the *Smo*-deficient TMJ.

#### 5.2.5. Summary

Taken together, these findings support the importance of IHH-mediated suppression of GLI3 and activation of GLI2 in the *Ihh* null MC-associated phenotype. However, the *Ihh^−/−^*;*Gli3^−/−^* mutants did not provide a full phenotypic rescue (also see AD section below). Nor did loss of *Gli2* alone entirely phenocopy the *Ihh* null phenotype. The fact that *Gli2* or *Gli3* null embryos exhibited non-identical phenotypes [58,63] in distinct tissues supports the possibility of distinct roles for different downstream effectors. These data also suggested a potential contribution of Hh pathway effectors other than GLI2/3, with non-canonical Hh pathway effectors being likely candidates [64,65,66]. Nevertheless, these data do support a significant contribution of GLI2 and GLI3-associated functions in the *Ihh* null phenotype.

### 5.3. Articular Disc Development

#### 5.3.1. Indian Hedgehog (*Ihh*) and Glioma-associated Oncogene-3 (*Gli3*)

*Ihh* null animals lacked both an AD or a lower articular cavity [32]. This was accompanied by loss of Hh signalling activity, as determined by low or absent *Gli1*, *Gli2* and *Ptch1* expression in the AD precursor mesenchyme. The lack of AD formation and joint cavitation in the *Ihh* null was not due to the unrestrained activity of GLI3 as the *Ihh*, *Gli3* double germline null also failed to develop an AD or undergo correct cavitation [17]. This developmental failure correlated with a failure of *Ihh* null and *Ihh^−/−^*;*Gli3^−/−^* animals to effectively recover expression of core Hh signalling components (*Ptch1* and *Gli2*) or indicators of pathway activity (*Ptch1* and *Gli1*) within the AD-associated regions. Expression of genes involved in Hh perception and signal transduction in the normal AD precursor mesenchyme supports the idea that MCC-derived IHH signals to the AD. Furthermore, a GLI3-independent role for IHH in AD morphogenesis is supported by the failure of the loss of *Gli3* function to rescue the *Ihh* null AD defects.

*Col2a1*-Cre-ERT2-mediated postnatal recombination of a conditional loss-of-function *Ihh* allele revealed thickening and partial fusion of the AD onto the articular surfaces of both the MC and GF [40]. Interestingly, expression of the synovial lubricating proteoglycan *Lubricin* was upregulated in the both the AD and the articular surface of the GF, potentially in compensation for improper AD function. In summary, postnatal *Ihh* signalling was required to complete the postnatal development of the AD and synovial cavities. Such findings are in agreement with postnatal developmental nature of the TMJ [7,8].

#### 5.3.2. Glioma-Associated Oncogene-2 (*Gli2*)

At E18.5 germline, *Gli2* mutants lacked a true articular disc, but harbored an abnormal cell layer attached to the articular surface of the MC [17]. In agreement with a more generalized role in fibrocartilaginous cartilage development, *Gli2* mutant intervertebral fibrocartilaginous discs also failed to develop [58,67]. Nevertheless, the presence of the abnormal cell layer in the location of the AD differed from the total absence observed in *Ihh* null animals [32]. It therefore appeared that impaired *Gli2* function contributed to, but could not entirely account for, the absence of an AD in the *Ihh* null mouse. These findings therefore support a role for some GLI2- independent signaling in AD development. An important follow up experiment would be to combine germline loss of *Ihh* with a gain of function *Gli2* allele in an attempt to rescue AD and cavitation defects.

#### 5.3.3. Smoothened (*Smo*)

*Sox9*-Cre;*Smo^fl/fl^* animals resulted in loss of *Smo* function in most, but not all, of the cells of the MC, AD and GF at around E17.5 [17]. In contrast to *Gli2* and *Ihh* null animals, *Sox9*-*Cre;Smo* mutant animals formed of an articular disc (AD) and an upper, but not a lower, articular cavity. As a result of the failed cavitation the AD remained attached to the MC. This less severe AD phenotype could be explained by technical differences between germline nulls and conditional Cre-mediated loss of function. One explanation resides in the retention of Smo signalling within (i) a small number of those *Sox9*-expressing cells that either failed to recombine the *floxed* *Smo* allele and/or (ii) a cell population derived from a non-*Sox9*-expressing lineage. More controversially, it could also indicate important contributions from non-canonical signalling upstream of *Smo* and downstream of *Ihh* [64,65,66]. Overall, these results partially supported a role for Smo-associated signalling in the maturation of the articular disc/cavity space.

#### 5.3.4. Summary

In summary, the combined findings of the *Ihh*, *Gli2*, *Gli3* and *Smo* loss of function studies all supported an essential role for Hh-signalling in correct AD and cavity formation and maturation. However, there were differences between the various mutant phenotypes and it appeared that GLI2 was more important than GLI3 in AD development and cavitation.

### 5.4. Other Genes Associated with Aberrant Hedgehog Signalling and TMJ Defects 

#### 5.4.1. Intraflagellar Transport (*IFT*) Genes

Although not considered *bona fide* members of the pathway, a number of cilia-associated genes/proteins influence Hh pathway activity. In vertebrates, Hh signalling acts through primary cilia [68] and cilia-associated intraflagellar transport (IFT) components regulate Hh signaling [69]. IFT components function by governing the transport of Hh signalling components into and out of the primary cilia. Within the MCC, primary cilia are present on the fibrous, polymorphic cells as well as flattened and hypertrophic chondrocytes [70]. In agreement with a role for cilia-regulated Hh signalling, *Ptch1* and *Gli1* are also expressed in both polymorphic cells and flattened cells (Figure 3A,B).

*Col2a1*-Cre-mediated loss of the IFT component *Kinesin-like protein-3* (*Kif3a*) led to a loss of primary cilia and a TMJ phenotype similar to Hh-deficient phenotypes [70]. *Kif3a* deficient animals exhibited malformed mandibles, an irregular bony surface, fusion of the AD onto the MC and improper joint cavitation. Further similarities included a disorganised MCC, reduced cell proliferation, increased numbers of hypertrophic chondrocytes and reduced *Sox9* expression. 

Surprisingly, *Ihh* expression was upregulated within the hypertrophic chondrocytes and a concomitant increase in *Ptch1* expression in the adjacent polymorphic chondroprogenitor layer and perichondrium. Hence, loss of IFT function resulted in increased *Ihh* ligand expression and evidence of increased Hh pathway activity. However, it also manifested a phenotype very similar to that of *Ihh* nulls. Such results would argue for a role for increased, rather than decreased, Hh signalling in promoting the observed MC defects. It is uncertain whether the changes in Hh signalling landscape were causal or consequential in nature. For example, the increase in expression of *Ihh* ligand and its cognate receptor *Ptch1*, may reflect a compensatory homeostatic response to increase the IFT-mediated deficit in Hh signalling. Further investigation of the Hh signalling landscape and understanding crosstalk with other signaling pathways may help resolve this apparent paradox.

The role of IFT in positively or negatively regulating Hh signalling can be contradictory as the action of IFT affect the generation of both activator and repressor forms of GLI proteins [68,71,72]. Hence, loss of IFT function can induce a loss or gain of Hh-associated phenotype in different cell types/tissues based on their particular reliance on either GLI3R or GLI activators. For example, GLI activators have a predominant role in neural patterning, while GLI3R has a predominant role in early limb development [68,71,72].

#### 5.4.2. Sry-related HMG box 9 (*Sox9*) and Runt-related Transcription Factor-2 (*Runx2*)

Both *Sox9* and *Runx2* play important roles in regulating chondrogenesis in long bones [52]. In the TMJ, loss of either *Sox9* [31,73] or *Runx2* [74] resulted in reduced Hh signalling and TMJ developmental defects. Loss of *Runx2* resulted in a failure to form the MCC as well as other secondary cartilage. However primary cartilage was not affected, thereby highlighting distinctions between chondrogenesis of primary and secondary cartilage. As with loss of *Runx2*, loss of *Sox9* function also resulted in the absence of the MCC as well as impaired GF formation and AD-associated cavitation [73].

Interestingly, *Runx2* can directly regulate *Ihh* transcription through binding *Ihh* promoter/enhancer elements [75,76] thereby placing it upstream of *Ihh* expression and Hh signalling. However, the epistatic relationship between Hh signalling and *Runx2* is a complex one as in the TMJ *Ihh* was required for *Runx2* expression [32,40]—thereby placing *Ihh* upstream of *Runx2*. Tissue specific differences could account for these discrepancies in addition to the existence of positive/negative feedback mechanisms. For example, *Runx2*-mediated activation of Hh signalling could feed forward to further enhance the activity of *Runx2*. In such a scenario, loss of either component results in the loss/decrease in the activity of the other.

#### 5.4.3. Short Stature Homeobox Gene 2 (*Shox2*)

The homeobox gene *short stature homeobox gene 2* (*Shox2*) also appears to influence Hh signalling. *Wnt1*-Cre-mediated conditional loss of *Shox2* resulted in the delayed development and subsequent dysplasia of the MCC and GF, as well as a failure in AD-associated cavitation [34]. *Shox2*-deficient tissue exhibited a reduction in *Ihh* expression in the developing MCC and ectopic overexpression in the developing GF. These results suggested that SHOX2 could promote the magnitude of *Ihh* expression in the MCC and spatio-temporally suppress *Ihh* expression in the GF. It is currently unknown whether these effects were direct/cell autonomous or indirect/non-cell-autonomous. Although the underlying mechanism of SHOX2 is unclear, it is interesting to note that it can act as both a transcriptional activator and repressor [77].

#### 5.4.4. Trichorhinophalangeal Syndrome 1 (*Trps1*)

Another noteworthy mouse model revealed that germline loss of *Trichorhinophalangeal syndrome 1* (*Trps1*) phenocopied many aspects of the *Ihh* null phenotype. *Trps1* null animals exhibited an undersized MC, no AD development and a lack of cavity formation [78]. In contrast to *Ihh* nulls, TRPS1 null animals exhibited an expansion of the *Runx2*/*Sox9* expression domains and increased magnitude of *Ihh* expression within the developing MCC. Surprisingly, these molecular effects are the exact opposite of the molecular effects observed in the *Ihh* null TMJ. Hence, it appears that very similar morphological outcomes can be associated with either (i) elevated *Ihh*, *Runx2* and *Sox9* expression (*Trps1* null) or (ii) decreased *Ihh* and *Runx2,* and *Sox9* expression (*Ihh* null).

#### 5.4.5. Summary

These findings reinforce the difficulty in the binary assignment of genes as being either suppressors or promoters of *Ihh* ligand expression or Hedgehog pathway activity. Furthermore, they confound our ability to assign binary roles for Hh signalling in either promoting or inhibiting correct TMJ development. Rather it appears to be highly context specific and/or held in a delicate balance; too little or too much cement, in the wrong place or at the wrong time, can make a structure unstable.

## 6. Perspectives

The development of the TMJ is a fascinating process that is intimately linked to Hh signalling. Future studies will undoubtedly continue to highlight the TMJ as a paradigm of intra- and inter-tissue Hh signalling in embryonic and postnatal tissues. In general, the correct development of the MCC, AD and GF all appear to be critically dependent on appropriate spatial, temporal expression as well as the magnitude of IHH-mediated Hh signalling.

One cautionary note about the state of our current knowledge centres on the heavy reliance on semi-quantitative analysis of mRNA expression and in situ hybridization data. 3D-based data acquisition of both mRNA and protein combined with appropriate image-based quantitation methods will be beneficial. As would complementary quantitative methods such as luciferase- or fluorescence-based in vivo activity reporters, immunofluorescence and laser-capture micro-dissection coupled with qRT-PCR. Together, these efforts will help establish a more accurate molecular profile of the Hh signalling landscape within the TMJ. As in other organs and tissues, the development of in vitro organoid models [79] should also facilitate interrogation of the molecular events during TMJ development.

Two important elements currently missing from our understanding of TMJ development are (i) the signal transductions pathways that initiate, maintain and restrain *Ihh* expression and (ii) the potential contribution of non-canonical Hh signalling [64,65,66]. Going forward, it will be important to apply consistent methodological means to perturb gene function and analyse the outcomes. Only then will we be able to better understand the contributions Hh signalling has in the development, maturation and maintenance of this intriguing synovial joint.
